# Characterisation of type 2 diabetes subgroups and their association with ethnicity and clinical outcomes: a UK real-world data study using the East London Database

**DOI:** 10.3399/BJGP.2021.0508

**Published:** 2022-05-17

**Authors:** Rohini Mathur, Sally A Hull, Sam Hodgson, Sarah Finer

**Affiliations:** Institute for Population Health Sciences, Barts and the London School of Medicine and Dentistry, Queen Mary University of London, London, and Department of Non-Communicable Disease Epidemiology, London School of Hygiene and Tropical Medicine, London.; Institute for Population Health Sciences, Barts and the London School of Medicine and Dentistry, Queen Mary University of London, London.; Primary Care Research Centre, University of Southampton, Southampton, UK; Institute for Population Health Sciences, Barts and the London School of Medicine and Dentistry, Queen Mary University of London, London.

**Keywords:** epidemiology, inequalities, primary care, type 2 diabetes

## Abstract

**Background:**

Subgroups of type 2 diabetes (T2DM) have been well characterised in experimental studies. It is unclear, however, whether the same approaches can be used to characterise T2DM subgroups in UK primary care populations and their associations with clinical outcomes.

**Aim:**

To derive T2DM subgroups using primary care data from a multi-ethnic population, evaluate associations with glycaemic control, treatment initiation, and vascular outcomes, and to understand how these vary by ethnicity.

**Design and setting:**

An observational cohort study in the East London Primary Care Database from 2008 to 2018.

**Method:**

Latent-class analysis using age, sex, glycated haemoglobin, and body mass index at diagnosis was used to derive T2DM subgroups in white, South Asian, and black groups. Time to treatment initiation and vascular outcomes were estimated using multivariable Cox-proportional hazards regression.

**Results:**

In total, 31 931 adults with T2DM were included: 47% South Asian (*n* = 14 884), 26% white (*n* = 8154), 20% black (*n* = 6423). Two previously described subgroups were replicated, ‘mild age-related diabetes’ (MARD) and ‘mild obesity-related diabetes’ (MOD), and a third was characterised ‘severe hyperglycaemic diabetes’ (SHD). Compared with MARD, SHD had the poorest long-term glycaemic control, fastest initiation of antidiabetic treatment (hazard ratio [HR] 2.02, 95% confidence interval [CI] = 1.76 to 2.32), and highest risk of microvascular complications (HR 1.38, 95% CI = 1.28 to 1.49). MOD had the highest risk of macrovascular complications (HR 1.50, 95% CI = 1.23 to 1.82). Subgroup differences in treatment initiation were most pronounced for the white group, and vascular complications for the black group.

**Conclusion:**

Clinically useful T2DM subgroups, identified at diagnosis, can be generated in routine real-world multi-ethnic populations, and may offer a pragmatic means to develop stratified primary care pathways and improve healthcare resource allocation.

## INTRODUCTION

Type 2 diabetes (T2DM) is a heterogeneous, multifactorial condition with a major impact on the health of global populations and with a high economic cost, largely mediated through its vascular complications.^1^ Recent studies have identified distinct and replicable subgroups of patients with T2DM in both experimental^2^^–^^4^ and non-experimental (real-world) cohorts^5^^–^^7^^,^^8^ using data-driven clustering methods. These studies have defined T2DM subgroups using clinical variables typically assessed in secondary care settings, including measures of insulin secretion and resistance (determined using C-peptide and glucose assays), to delineate subgroups and their likely aetiology.^2^^–^^4^^,^^7^ However, in the UK, the majority of T2DM management takes place in primary care settings, where these investigations are rarely performed. It is not known whether using data routinely available in the primary care setting can also be used to identify T2DM subgroups and their association with clinical outcomes. The impact of ethnicity on the characterisation of T2DM subgroups is also poorly understood but is an important area of study given the varied aetiological processes and disease prevalence, and outcomes seen in different ethnic groups with the condition.^9^^–^^11^

The current study therefore set out to characterise T2DM subgroups in a large UK-based multi-ethnic population using routinely collected clinical measures captured in the primary care record. It then sought to investigate differences in control of glycated haemoglobin (HbA_1c_), time to initiation of antidiabetic treatment, and risk of vascular outcomes by diabetes subgroup and ethnicity. In doing so, the potential utility of data-driven T2D subgroup identification to stratify care delivery in NHS primary care was evaluated.

## METHOD

### Study population

An observational cohort study was conducted using the East London Primary Care Database, which includes anonymised longitudinal health record data from 1 million individuals registered at 125 general practices across the three multi-ethnic Inner London boroughs of Tower Hamlets, Newham, and Hackney (http://www.blizard.qmul.ac.uk/ceg-home.html).The study population included all adults aged ≥18 years newly diagnosed with T2DM between January 2008 and January 2018 with at least 12 months of continuous registration before first diagnosis of T2DM. T2DM diagnosis was identified using the C10F% Read codes as defined in the UK Quality and Outcomes Framework (see Supplementary Table S1 for the codelists).^12^ Study follow-up commenced on the date of T2DM diagnosis and ended at the earliest of leaving the GP practice, death, or 1 January 2018.

**Table:** How this fits in

Previous studies of predominantly white European populations have identified four type 2 diabetes subgroups. In the UK the clinical measures necessary to replicate these subgroups are only available in secondary care data, limiting their usefulness for diabetes management in primary care settings. The current study demonstrated how clinically meaningful type 2 diabetes subgroups can be pragmatically generated using real-world primary care data. Furthermore, it highlighted important differences between type 2 diabetes subgroups with respect to vascular outcomes, treatment initiation, and glycated haemoglobin control. Diabetes subgroups are a useful heuristic for assisting decision making by clinicians that, in turn, can lead to a more personalised design of diabetes care focused on more intensive management of subgroups most at risk of complications, such as those with severe hyperglycaemia at time of diagnosis.

### Covariates

Self-reported ethnicity was identified using Read codes and collapsed into the five high-level categories of the 2011 Census (white, South Asian, black African/Caribbean, Mixed, and other) (Supplementary Table S1). Age at T2DM diagnosis was calculated from the date of diagnosis and age at data extraction (January 2018). Deprivation was measured using the Townsend score and divided into quintiles. Baseline was defined as the date of T2DM diagnosis. Baseline measures of HbA_1c_, body mass index (BMI), systolic and diastolic blood pressure, total cholesterol, and estimated glomerular filtration rate (eGFR) were derived from the last value in the year before diagnosis. Comorbid conditions were considered prevalent at baseline if present on the clinical record at any date before T2DM diagnosis. These included hypertension, coronary heart disease (CHD), stroke, heart failure, chronic kidney disease (CKD), retinopathy, and neuropathy.

### Outcomes

HbA_1c_ in the 5 years following initial diagnosis was calculated for each individual by taking the mean of all HbA_1c_ values recorded in each 12-month period. Antidiabetic medications were categorised as all oral non-insulin antidiabetic drugs and insulin. Macrovascular disease was defined as a composite of CHD, heart failure, myocardial infarction, and stroke. Microvascular disease was defined as a composite of CKD, neuropathy, and retinopathy. Incident macrovascular and microvascular events were defined as diagnoses recorded at any point after T2DM diagnosis.

### Statistical analysis

Latent-class analysis was used to identify subgroups of T2DM for the whole cohort and separately for individuals of white, South Asian, and black ethnicity. Subgroups were derived using data from four observed indicator variables: age at diagnosis, sex, HbA_1c_ at diagnosis, and BMI at diagnosis. Models with between two and five classes were compared and the optimal number of classes was chosen by evaluating the Bayesian Information Criteria (BIC, with lower values indicating better fit), clinical interpretability, minimum posterior probability of group membership over 70%, and sufficient group membership, defined as >1% of the study population in each class.

Mean HbA_1c_ in the 5 years following initial diagnosis was plotted over time by ethnic group and T2DM subgroup. Among those free from prevalent vascular disease at baseline, age- and sex-adjusted Cox-proportional hazards regression was used to estimate differences in the cause-specific risk of incident macro- and microvascular disease between T2DM subgroups by ethnic group. Among individuals free from any antidiabetic treatment at diagnosis, differences in time to initiation of antidiabetic treatment between subgroups for the whole population, and by ethnic group, were modelled using age- and sex-adjusted multivariable Cox-proportional hazards regression. Individuals who initiated treatment in the 30 days before diagnosis were included in the analysis by moving their treatment initiation date to 1 day after T2DM diagnosis as they were considered to be ‘baseline initiators’, for whom the initial prescription formed part of the diabetes diagnosis process.

## RESULTS

A total of 31 931 adults with T2DM were included in the study, of whom 47% were of South Asian ethnicity (*n* = 14 884), 26% were of white ethnicity (*n* = 8154), 20% were of black ethnicity (*n* = 6423), and 6% were of mixed or other ethnicities (*n* = 1957). Ethnicity was unknown for 1.6% of the study population (*n* = 513). A three-group latent-class model was chosen because of minimal improvement in BIC criteria or clinical interpretability when compared with four- and five-group models, and this was unchanged when the analysis was stratified by ethnicity. Maximum follow-up time in this study cohort was 10 years, with median follow-up time at 2.9 years (interquartile range 1.0–5.4 years).

### T2DM subgroups

**Figure 1.**
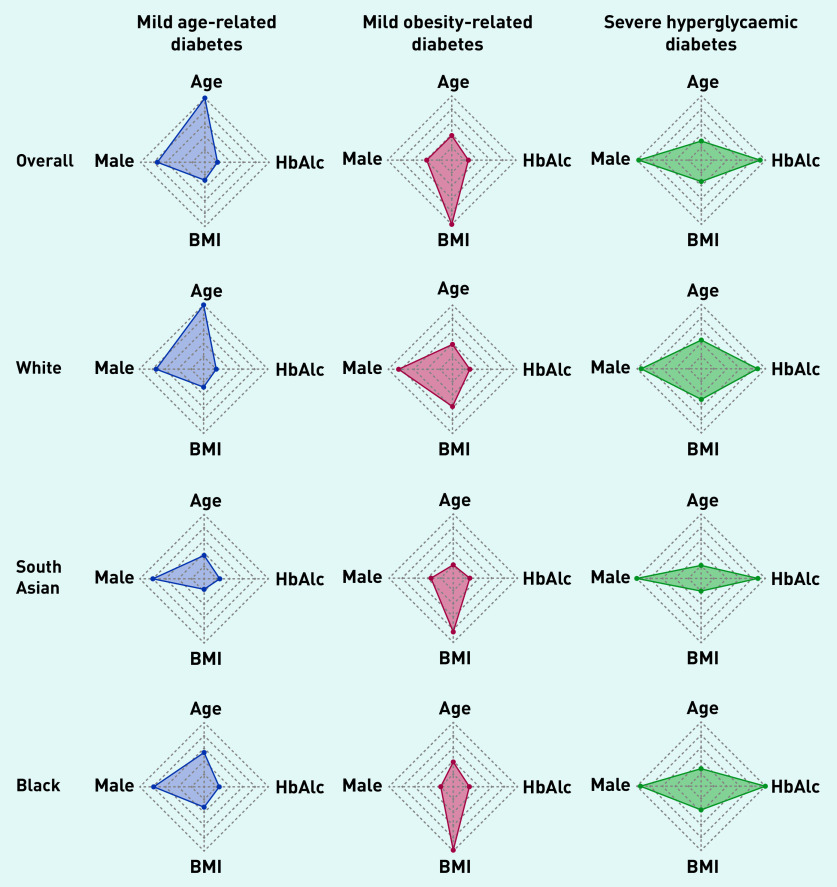
***Radar plots to show distribution of age, BMI, HbA1c, and sex (presented as proportion of males) across latent-class-derived clusters in the overall cohort and by ethnicity.*** *^a^* *^a^****The centre of each polygon represents the minimum value for that variable across all groups; the edge represents the maximum value. All polygons are plotted to the same scale. Overall cohort is: 82.3% MARD, 9.6% MOD, 8.1% SHD; South Asian ethnicity is 84.2% MARD, 8.5% MOD, 7.3% SHD; white ethnicity is 37.7% MARD, 53.7% MOD, 8.6% SHD; black ethnicity is 76.2% MARD, 13.4% MOD, 10.4% SHD. BMI = body mass index. HbA****_1c_* ***= glycated haemoglobin. MARD = mild age-related diabetes. MOD = mild obesity-related diabetes. SHD = severe hyperglycaemic diabetes.***

Across the whole population, in this study two T2DM subgroups were characterised that had been identified in previous studies: ‘mild age-related diabetes’ (MARD) was driven by age at diagnosis and was the most prevalent cluster (82% of the total population, *n* = 26 294), and ‘mild obesity-related diabetes’ (MOD) was driven by BMI at onset (10%, *n* = 3059) (Figure 1 and Table 1). The current study also identified a third subgroup, characterised by severe hyperglycaemia (determined by HbA_1c_) at diagnosis — ‘severe hyperglycaemic diabetes’ (SHD) — and this was the least prevalent cluster (8%, *n* = 2578).

**Table 1.** Baseline characteristics by T2DM subgroup for whole East London population and separately by ethnic group

**Characteristic**	**Overall**				**South Asian**				**White**				**Black**				**Data complete,^a^ %**
			
	**Overall**	**MARD**	**MOD**	**SHD**	**All South**	**MARD**	**MOD**	**SHD**	**All white**	**MARD**	**MOD**	**SHD**	**All black**	**MARD**	**MOD**	**SHD**	
					**Asian**												
*n*	31 931	26 294	3059	2578	14 884	12 538	1261	1085	8154	3075	4379	700	6423	4895	858	670	

**Latent-class predictors**																	
Age at diagnosis, years, mean (SD)	53.1 (13.8)	53.9 (13.9)	50.6 (12.8)	47.7 (12.1)	49.2 (13.1)	50.1 (13.1)	44.9 (12.4)	44.1 (11.2)	58.8 (13.5)	71.7 (8.2)	50.7 (9.1)	53 (11.7)	54.4 (13.0)	56 (12.8)	50.7 (11.8)	46.9 (12.3)	100
Male, %	17 271 (54.1)	14 793 (56.3)	698 (22.8)	1780 (69.0)	8136 (54.7)	7095 (56.6)	247 (19.6)	794 (73.2)	4674 (57.3)	1581 (51.4)	2624 (59.9)	469 (67.0)	3163 (49.2)	2649 (54.1)	64 (7.5)	450 (67.2)	100
HbA_1c_ at diagnosis, %, mean (SD)	7.8 (2)	7.2 (0.9)	7.3 (1.1)	12.1 (1.6)	7.8 (1.8)	7.2 (0.9)	7.3 (1.1)	11.8 (1.5)	7.8 (2.0)	6.9 (0.7)	7.3 (1.0)	11.9 (1.5)	8.0 (2.3)	7.2 (0.9)	7.3 (1.1)	12.8 (1.8)	60.3
HbA_1c_ at diagnosis, mmol/mol, mean (SD)	62.2 (21.7)	54.8 (10.1)	56.6 (12.1)	108.7 (17.8)	61.2 (19.8)	55.0 (9.9)	56.3 (11.8)	105.3 (16.8)	62.0 (21.6)	51.8 (8.2)	56.1 (10.7)	106.2 (16.8)	64.2 (25.3)	54.7 (10.4)	56.3 (11.9)	115.9 (19.9)	60.3
BMI at diagnosis, kg/m^2^, mean (SD)	30.4 (5.7)	28.9 (4.2)	41.3 (3.4)	30.2 (5.6)	28.7 (4.8)	27.6 (3.5)	38.5 (3.5)	28.1 (4.5)	32.5 (6.1)	29.5 (4.7)	34.6 (6.1)	32.7 (6.4)	31.7 (5.7)	30.0 (4.1)	41.1 (3.6)	30.9 (5.7)	89.1

**Deprivation score, %**																	
Deprivation Q1 (least deprived)	6399 (20.1)	5341 (20.4)	528 (17.3)	530 (20.7)	3728 (25.1)	3062 (24.5)	365 (29.0)	301 (27.8)	1320 (16.3)	504 (16.5)	692 (15.9)	124 (17.8)	904 (14.1)	702 (14.4)	114 (13.4)	88 (13.2)	100
Q2	6363 (20.0)	5206 (19.9)	608 (20.0)	549 (21.4)	3035 (20.4)	2539 (20.3)	251 (19.9)	245 (22.6)	1534 (18.9)	549 (17.9)	840 (19.3)	145 (20.8)	1286 (20.1)	997 (20.4)	160 (18.8)	129 (19.4)	100
Q3	6334 (19.9)	5261 (20.1)	627 (20.6)	446 (17.4)	2668 (18.0)	2278 (18.2)	230 (18.3)	160 (14.8)	1850 (22.8)	754 (24.6)	964 (22.1)	132 (18.9)	1324 (20.7)	1013 (20.8)	178 (20.9)	133 (20.0)	100
Q4	6482 (20.4)	5261 (20.1)	676 (22.2)	545 (21.3)	2849 (19.2)	2423 (19.4)	224 (17.8)	202 (18.7)	1711 (21.1)	620 (20.2)	924 (21.2)	167 (23.9)	1436 (22.4)	1074 (22.0)	199 (23.3)	163 (24.5)	100
Q5 (most deprived)	6230 (19.6)	5129 (19.6)	607 (19.9)	494 (19.3)	2562 (17.3)	2199 (17.6)	189 (15.0)	174 (16.1)	1705 (21.0)	636 (20.8)	939 (21.5)	130 (18.6)	1448 (22.6)	1093 (22.4)	202 (23.7)	153 (23.0)	100

**Comorbid conditions at diagnosis, %**																	
Any macrovascular	3118 (9.8)	2730 (10.4)	242 (7.9)	146 (5.7)	1210 (8.1)	1096 (8.7)	67 (5.3)	47 (4.3)	1260 (15.5)	773 (25.1)	418 (9.5)	69 (9.9)	420 (6.5)	346 (7.1)	51 (5.9)	23 (3.4)	100
Any microvascular	1573 (4.9)	1372 (5.2)	154 (5.0)	47 (1.8)	500 (3.4)	457 (3.6)	34 (2.7)	9 (0.8)	606 (7.4)	478 (15.5)	106 (2.4)	22 (3.1)	375 (5.8)	314 (6.4)	44 (5.1)	17 (2.5)	100

**Clinical characteristics at diagnosis, mean (SD)**																	
Systolic blood pressure, mmHg	134.7 (18.3)	134.5 (18.4)	136.6 (18.0)	134.7 (18.3)	131.2 (17.5)	131.1 (17.6)	131.9 (16.3)	131.1 (17.4)	137.6 (18.0)	138.2 (17.8)	137.0 (18.1)	137.8 (17.7)	138.5 (19.1)	138.8 (19.2)	137.9 (18.4)	137.2 (19.7)	86.3
Diastolic blood pressure, mmHg	81.5 (12.6)	81.0 (12.8)	83.7 (11.4)	83.0 (11.4)	80.5 (13.6)	80.2 (14.0)	82.9 (11.2)	81.8 (10.7)	81.8 (11.5)	78.0 (10.8)	84.4 (11.2)	84.1 (11.5)	82.9 (11.7)	82.5 (11.7)	84.5 (11.9)	83.9 (11.9)	86.3
Total cholesterol, %	5.2 (2.7)	5.1 (2.6)	5.2 (1.1)	5.7 (4.7)	5.2 (3.2)	5.1 (3.5)	5.0 (1.0)	5.6 (1.4)	5.2 (2.9)	4.8 (1.2)	5.4 (1.4)	6.0 (8.6)	5.2 (1.2)	5.2 (1.2)	5.2 (1.1)	5.6 (1.5)	83.8
eGFR, ml/min	80.6 (16.5)	80.3 (16.6)	80.9 (16.9)	82.9 (15.1)	84.3 (15.5)	83.7 (15.5)	87.6 (16.3)	87.7 (12.8)	77.5 (17.5)	69.9 (17.3)	83.2 (15.5)	81.1 (16.0)	74.5 (15.5)	74.0 (15.6)	76.0 (14.7)	76.7 (15.3)	72.1

a *Percentage of the data for the entire population that are complete. eGFR = estimated glomerular filtration rate. HbA_1c_ = glycated haemoglobin. MARD = mild age-related diabetes. MOD = mild obesity-related diabetes. Q = quintiles. SD = standard deviation. SHD = severe hyperglycaemic diabetes.*

### Ethnic differences in T2DM subgroup membership

Next, how cluster membership and clinical features varied by ethnicity (Figure 1 and Table 1) was explored. In the white population, the MOD subgroup was the commonest (54% of individuals, [4379/8154]) and was characterised by severe obesity (mean BMI 34.6 kg/m^2^); 38% (3075/8154) of the white population fell into the MARD subgroup (mean age 71.7 years), and 9% (700/8154) fell into the SHD subgroup (mean HbA_1c_ 11.9%/106.2 mmol/mol). In contrast, among South Asians, the MARD subgroup was the commonest (84% [12 538/14 884]) but was driven by a much younger mean age than in the white population (50.1 years). The MOD and SHD subgroups were uncommon (8% [1261/14 884] and 7% [1085/14 884], respectively) in South Asian people.

As shown in Figure 1, the pattern and features of subgroups seen in South Asians were mirrored in the black African Caribbean population, with 76% (4895/6423) having MARD (mean age 56.0), 13% (858/6423) with MOD, and 10% (670/6423) with SHD. The clinical features driving MOD (BMI 41.4 kg/m^2^) and SHD (mean HbA_1c_ 12.8%/115.9 mmol/mol) were more extreme in black African Caribbean groups than in white and South Asian groups (Table 1).

In both white and black ethnic groups, the proportion of people with T2DM increased with increasing levels of deprivation. However, this gradient was reversed in the South Asian group with the majority of individuals contributing to the least deprived quintile. Although the sex split was comparable between ethnic groups for the MARD and SHD subgroups, the MOD subgroup was 40% female for the white group, 80.4% female for the South Asian group, and 92.5% female in the black group (Table 1).

### HbA_1c_ trajectories over time

**Figure 2.**
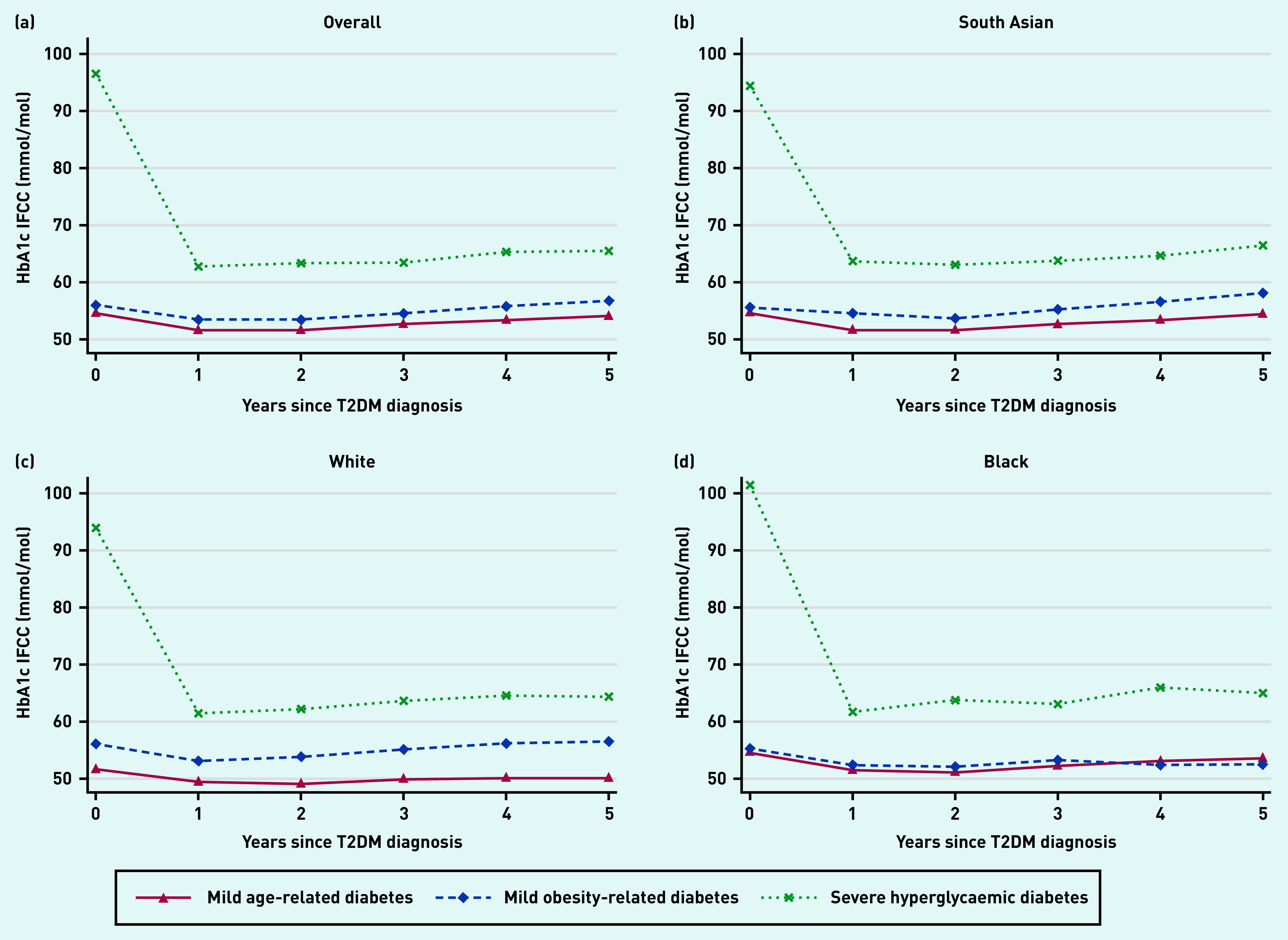
*HbA_1c_ trajectories by latent subgroup and ethnic group. a) Overall; b) South Asian; c) white; and d) black ethnicity. HbA_1c_ = glycated haemoglobin. IFCC = International Federation of Clinical Chemistry and Laboratory Medicine. T2DM = type 2 diabetes.*

In the MARD and MOD subgroups, HbA_1c_ was below 7.6%/60 mmol/mol at diagnosis and remained so over the first 5 years of follow-up. In the SHD subgroup, HbA_1c_ was significantly elevated at diagnosis (>12.7%/90 mmol/mol) and brought down rapidly within the first 12 months, although never achieving target control. Patterns of HbA_1c_ trajectories in each of the ethnic groups mirrored that of the overall population (Figure 2).

### Time to vascular outcomes

**Figure 3.**
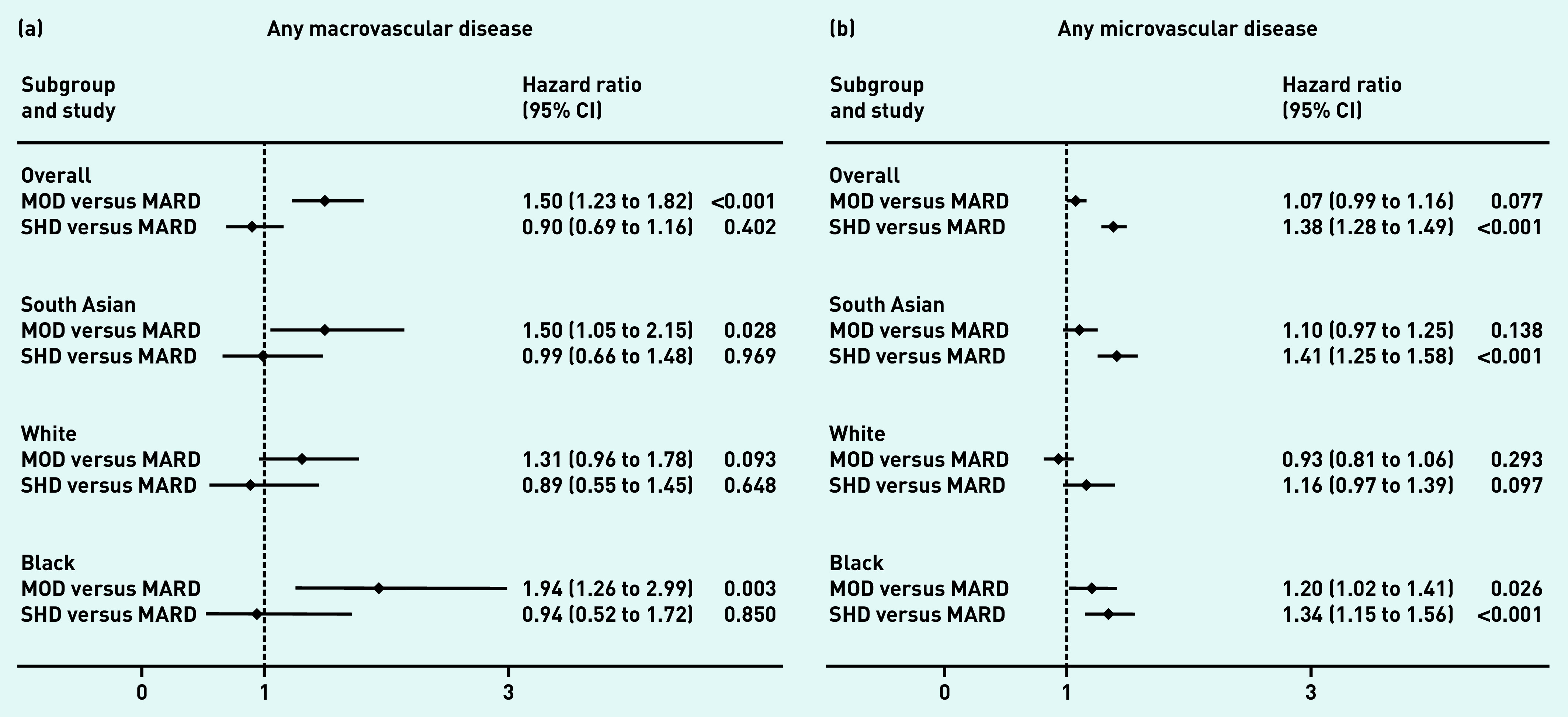
***Risk of incident vascular complications by T2DM subgroup and ethnic group among those free of complications at baseline. a) Macrovascular: any of cardiovascular disease, stroke, and heart failure. b) Microvascular: any of retinopathy, neuropathy, and chronic kidney disease.*** *^a^* *^a^* ***All models adjusted for age and sex. CI = confidence interval. MARD = mild age-related diabetes. MOD = mild obesity-related diabetes. SHD = severe hyperglycaemic diabetes. T2DM = type 2 diabetes mellitus.***

Among those free from vascular disease at baseline, 4.3% (1094/25 447) developed macrovascular complications (*n* = 1094) and 28.5% (7557/26 484) developed microvascular complications during follow-up (Supplementary Table S2). In comparison with the MARD subgroup, time to development of macrovascular disease was significantly increased in the MOD subgroup (hazard ratio [HR] 1.50, 95% confidence interval [CI] = 1.23 to 1.82), whereas time to development of microvascular disease was significantly increased in the SHD subgroup (HR 1.38, 95% CI = 1.28 to 1.49) (Figure 3).

These differences were most pronounced in the South Asian and black groups, for whom subgroup differences in risk of microvascular and macrovascular outcomes were similar. For individuals of white ethnicity, no differences in the risk of either micro- or macrovascular disease by subgroup were evident.

Despite the significant variation in age at onset across the MARD subgroups between the three ethnic categories, there was no differential association between MARD subgroup membership and vascular outcomes by ethnicity.

### Time to antidiabetic treatment initiation

Among those free from treatment at baseline, 94% (1954/2111) of those in the SHD subgroup, 82% (2016/2455) of those in the MOD subgroup, and 77% (16 781/21 860) of those in the MARD subgroup initiated non-insulin antidiabetic treatment, whereas 11.4% (251/2202) of those in the SHD subgroup, 6.5% (169/2617) of those in the MOD subgroup, and 5.2% (1170/22 623) of those in the MARD subgroup-initiated insulin treatment.

After adjustment for age and sex, initiation of non-insulin and insulin antidiabetic treatment was twice as fast in the SHD subgroup in comparison with the MARD subgroup (non-insulin HR 1.92, 95% CI = 1.83 to 2.02; insulin HR 2.02, 95% CI = 1.76 to 2.32), with differences between the MOD and MARD subgroups smaller in magnitude (non-insulin HR 1.16, 95% CI = 1.11 to 1.22; insulin HR = 1.02, 95% CI = 0.87 to 1.21). Subgroup differences in age- and sex-adjusted time to treatment initiation were largest in the white population and smallest in the black population (Table 2).

**Table 2.** Treatment initiation characteristics by diabetes subgroup and ethnicity

**Population**	**Non-insulin oral antidiabetic medications**				**Insulin**			
	**Number eligible to initiate**	**Initiating treatment, *n* (%)**	**Years to initiation, mean (SD)**	**Relative risk (95% CI),^a^ *P*-value**	**Number eligible to initiate**	**Initiating treatment, *n* (%)**	**Years to initiation, mean (SD)**	**Relative risk (95% CI),^a^ *P*-value**
**Overall**	26 426	20 781 (78.6)	0.9 (1.7)		27 442	1590 (5.8)	3.7 (2.7)	
MARD	21 860	16 781 (76.8)	1.0 (1.7)	1 (ref)	22 623	1170 (5.2)	3.7 (2.7)	1 (ref)
MOD	2455	2016 (82.1)	0.8 (1.6)	1.16 (1.11 to 1.22), <0.001	2617	169 (6.5)	3.8 (2.7)	1.02 (0.87 to 1.21), 0.778
SHD	2111	1984 (94.0)	0.3 (1.1)	1.92 (1.83 to 2.02), <0.001	2202	251 (11.4)	3.4 (2.6)	2.02 (1.76 to 2.32), <0.001
**South Asian**	11 921	9832 (82.5)	0.7 (1.4)		12 445	563 (4.5)	3.7 (2.6)	
MARD	10 129	8196 (80.9)	0.8 (1.5)	1 (ref)	10 518	418 (4.0)	3.7 (2.6)	1 (ref)
MOD	933	804 (86.2)	0.6 (1.3)	1.14 (1.06 to 1.23), <0.001	1017	60 (5.9)	3.6 (2.5)	0.98 (0.74 to 1.29), 0.879
SHD	859	832 (96.9)	0.1 (0.7)	2.05 (1.90 to 2.20), <0.001	910	85 (9.3)	3.5 (2.5)	2.12 (1.67 to 2.69), <0.001
**White**	7076	5256 (74.3)	1.1 (2)		7307	468 (6.4)	3.9 (2.7)	
MARD	2734	1714 (62.7)	1.5 (2.2)	1 (ref)	2837	111 (3.9)	3.9 (2.7)	1 (ref)
MOD	3749	3000 (80.0)	0.9 (1.7)	1.40 (1.29 to 1.53), <0.001	3860	277 (7.2)	3.9 (2.8)	0.92 (0.68 to 1.25), 0.597
SHD	593	542 (91.4)	0.4 (1.3)	2.43 (2.17 to 2.72), <0.001	610	80 (13.1)	3.5 (2.6)	2.00 (1.41 to 2.83), <0.001
**Black**	5400	4152 (76.9)	0.9 (1.7)		5558	448 (8.1)	3.6 (2.7)	
MARD	4131	3078 (74.5)	1.0 (1.8)	1 (ref)	4245	312 (7.3)	3.6 (2.7)	1 (ref)
MOD	709	556 (78.4)	0.8 (1.6)	1.12 (1.02 to 1.23), 0.022	739	49 (6.6)	3.6 (2.7)	0.72 (0.53 to 0.99), 0.045
SHD	560	518 (92.5)	0.3 (1.2)	1.81 (1.64 to 2.00), <0.001	574	87 (15.2)	3.3 (2.6)	1.56 (1.21 to 2.00), <0.001

a *Age- and sex-adjusted relative risk versus MARD (reference group). CI = confidence interval. MARD = mild age-related diabetes. MOD = mild obesity-related diabetes. Ref = reference. SD = standard deviation. SHD = severe hyperglycaemic diabetes.*

## DISCUSSION

### Summary

In this observational cohort study, three T2DM subgroups were identified in an ethnically diverse UK population. Using only routinely recorded primary care clinical observations the current study replicated two subgroups previously reported in experimental and trial cohorts but this study was unable to identify subgroups based on insulin secretion or insulin resistance, as these rely on biomarkers not widely available in primary care settings.

The current study showed that T2DM subgroups defined at the time of diagnosis were strong predictors of clinically important differences in time to onset of vascular disease, time to initiation of antidiabetic treatment, and attainment of glycaemic control. People classified as having MARD at diagnosis had the slowest progression to vascular complications, slowest initiation of antidiabetic treatment, and best long-term glycaemic control. This association was observed consistently across all ethnic groups, despite age at diagnosis being 22 and 16 years earlier in South Asian and black groups, respectively, than in the white group. Future research will need to investigate the longer-term impact of the MARD phenotype on vascular complications in South Asian and black populations

People classified as having MOD at diagnosis maintained target HbA_1c_ control over the study duration, had faster treatment initiation than those in the MARD subgroup, and had the fastest progression to macrovascular complications. Those classified as having SHD at diagnosis had persistently poor glycaemic control that never reached target thresholds and had the highest risk of microvascular outcomes despite having the fastest initiation of antidiabetic medication. These findings suggest that people in the age-related subgroup may require less intensive clinical care processes, but that those in the SHD subgroup are likely to benefit from enhanced monitoring, support, and management of their condition.

There were significant differences in the clinical features of the diabetes subgroups according to ethnicity. For example, the MOD subgroup had disproportionately more women from South Asian and black ethnicities than the white ethnic group. The age at diagnosis of people with MARD was 20 years younger in those of South Asian and black ethnicity compared with white ethnicity. South Asian and black people in the MOD and SHD subgroups had higher risk of vascular complications than those of white ethnicity in the MOD and SHD subgroups. Future research in larger multi-ethnic populations will be needed to further investigate the impact of sex and age at diabetes onset on disease outcomes.

### Strengths and limitations

The current study benefited from a significantly larger and more ethnically diverse population than many earlier studies used for identifying T2DM subgroups.^2^^,^^4^^,^^5^ Furthermore, ethnicity was recorded for 98% of the study population, ensuring that the ethnicity-specific T2DM analyses were unlikely to be biased. This study captured all people with T2DM registered within a contiguous geographic area representative of the general population and other urban centres in the UK that are also ethnically and socially diverse. Furthermore, all practices contributing to this study were following standard diabetes management guidelines as outlined by the National Institute for Health and Care Excellence^13^ and performance standards as per the Quality and Outcomes Framework.^14^

Measures of serum high-density lipoprotein and triglycerides were not included in the cluster analysis as these are not uniformly collected in routine primary care practice. Their inclusion would have added further refinement to diabetes subgroups as they are surrogate measures of insulin resistance. As a result of the small number of people of mixed ethnicity, there was not sufficient statistical power to generate reproducible latent classes in this ethnic group or estimate interpretable associations with vascular complications, treatment initiation, and HbA_1c_ trajectories. The East London Primary Care Database is subject to the same strengths and biases as all routine data.^15^ It is possible that, by using diagnostic codes to define diabetes, some individuals with type 1 diabetes may have been misclassified as having T2DM. Prescriptions for antidiabetic medications issued in primary care were identified in this study but it was not possible to determine whether prescriptions were filled or taken as indicated.

Finally, linked secondary care data were not available and acute vascular events coded in hospital settings only may have been missed.

### Comparison with existing literature

A 2020 systematic review of cluster-based approaches to diabetes subtypes identified 14 studies, of which the majority found identical clusters.^16^ First reported by Ahlqvist and colleagues in 2018, clusters related to T2DM included: ‘severe insulin deficient diabetes’, ‘severe insulin resistant diabetes’, MOD, and MARD.^4^ These subgroups have been replicated across a number of settings including the Netherlands and Scotland,^7^ Ukraine,^17^ China,^18^ and India.^5^ Only one included study was conducted in the UK; however, this was a cross-sectional hospital-based study of 33 children with type 1 diabetes.^19^

The subgroups identified in the current study closely align with those previously reported. The MARD and MARD subgroups in the current study resembled the MARD and MOD clusters in previous studies. The SHD cluster was specific to the current study and is likely to include the previously reported ‘severe insulin deficient diabetes’ and ‘severe insulin resistant diabetes’ clusters.

### Implications for research and practice

This study has demonstrated that pragmatic T2DM subgroups can be generated using real-world primary care data and these subgroups can identify important differences in clinical characteristics and vascular outcomes. These findings have wider generalisability to national and global populations. The identification of these subgroups provides a useful heuristic for characterising differences between patients at the population level, including in ethnically diverse populations. The identification of these subgroups at diagnosis could help move away from a ‘one size fits all’ care pathway and instead offer a stratified care pathway that is readily enabled by clinical data systems. This stratification could enhance the care of those most at risk of complications, and de-intensify care for those who are not. Opportunities to stratify care are particularly relevant in the context of healthcare services constrained in time and financial resources in which many people with T2DM, and clinicians managing their care, feel their care needs are not met.^20^ The ability to apply data-driven clustering to real-world data offers wider generalisability to other chronic diseases largely managed in primary care such as hypertension and CKD.

Important next steps are to reproduce these findings in other multi-ethnic populations, using larger sample sizes, longer follow-up duration, and lipid profile measures to reproduce these findings at scale. Subsequently, empirical evaluation of subgroup-stratified care using a cluster randomised controlled trial with long-term measurement of outcomes is likely to be necessary.

## Data

The data used for this study comprise anonymised clinical records derived from the East London Database. Only the authors have access to the study data. Codelists are available in Supplementary Table S1. Researchers should contact the Clinical Effectiveness Group at Queen Mary University of London to request access to the data.
